# Curcumin-Dragon’s blood-chitosan nanosuspension for dual forensic detection

**DOI:** 10.1038/s41598-026-51407-z

**Published:** 2026-05-20

**Authors:** Adel Al Fatease, B. Nagashubha, Umme Hani, Ali H. Alamri, Kiran Sai Maccha, Awaji Y. Safhi, Fahad AlQahtani, Sree Lakshmi Mallam, Sowmya Vallabhi, Yashmeen Banu Shaik, Veeresh Chakali

**Affiliations:** 1 Department of Pharmaceutics, College of Pharmacy, King Khalid University, Abha, 62529 Saudi Arabia; 2 Department of pharmaceutics, Raghavendra Institute of Pharmacy Education and Research K.R. Palli Cross, Chiyyedu, Ananthapuramu, Andhra Pradesh 515721 India; 3 Department of Pharmaceutics, College of Pharmacy, Jazan University, Jazan, Saudi Arabia; 4 Department of Pharmaceutical Sciences, College of Pharmacy, Umm Al-Qura University, Makkah, Saudi Arabia

**Keywords:** Forensic detection, Latent fingerprint, Bloodstain detection, Curcumin, Dragon’s blood resin, Chitosan nanosuspension, Ionic gelation, Box-Behnken design, Biochemistry, Biological techniques, Biotechnology, Chemistry, Materials science, Nanoscience and technology

## Abstract

Forensic identification of latent fingerprints and bloodstains is fundamental to criminal investigations, yet conventional methods often suffer from high toxicity, low sensitivity, and substrate dependency. This study aims to address these limitations by developing a novel, biocompatible, dual-function nanosuspension formulation. The nanosuspension was synthesized using the ionic gelation method, crosslinking curcumin and Dragon’s blood resin within a chitosan nanoparticle matrix using sodium tripolyphosphate (TPP). Formulation optimization was systematically performed using a Box-Behnken Design (BBD) to evaluate the impact of chitosan concentration, curcumin amount, and TPP concentration on particle properties. Forensic efficacy was assessed through fluorescence imaging and ridge detail analysis on both porous and non-porous substrates. The optimized formulation achieved highly favorable physicochemical properties, including an average particle size of 220.5 ± 5.4 nm, a stable negative zeta potential of – 31.2 ± 2.1 mV, and an encapsulation efficiency of 85.2 ± 3.1%. The nanosuspension demonstrated significant quantitative superiority in forensic application, yielding a ridge contrast ratio of 4.2 ± 0.3 compared to 2.1 ± 0.4 for standard powders. Strong fluorescence under UV illumination enabled clear visualization of fingerprints up to the 5th depletion and selective marking of bloodstains. Molecular docking confirmed a strong binding affinity of resin components with hemoglobin (8.2 kcal/mol, supporting the observed selective detection. The novel curcumin-Dragon’s blood resin-chitosan nanosuspension provides a non-toxic, highly sensitive, and substrate-versatile dual-function reagent. This formulation represents a significant advancement in forensic evidence visualization with substantial potential for practical field deployment.

## Introduction

Forensic science plays a pivotal role in criminal investigations by providing objective evidence to accurately identify individuals and reconstruct crime scenes. Among various forensic evidences, latent fingerprints and bloodstains serve as primary biometrics and biological markers that significantly aid law enforcement agencies in suspect identification and crime resolution. However, the detection and visualization of these evidences remain challenging due to their often faint, invisible, or degraded nature on diverse substrates encountered at crime scenes.

Conventional methods of latent fingerprint detection rely heavily on physical powders such as carbon black or aluminium powders^14^, which demand direct application to evidence surfaces. These powders pose significant toxicity risks to forensic personnel and may offer limited contrast, especially on multi-coloured or complex substrates, thereby hampering effective visualization. Similarly, traditional blood detection protocols often employ chemical reagents that, while sensitive, have drawbacks including potential damage to fragile biological residues and interference with subsequent analyses. Moreover, current forensic reagents predominantly focus on detecting either latent fingerprints or bloodstains individually. This separation necessitates multiple detection procedures, increasing time, cost, and risk to evidentiary integrity.

Advancements in nanotechnology present a transformative opportunity to surmount these limitations by enabling novel formulations that are highly sensitive, selective, and safer for forensic applications^1–4^. Recent progress in nanomaterial-based biosensing systems has enabled the discriminative diagnosis of complex biological markers, while signal-enhanced immunoassays continue to push detection limits for sensitive proteins. Furthermore, the integration of nanomedicine with traditional natural products presents new opportunities for targeted delivery and forensic evidence marking^36,37,40^. Natural bioactive compounds, such as curcumin a fluorescent polyphenol derived from turmeric^5,8^ and Dragon’s blood resin an adhesive natural exudate possessing chromogenic properties have individually demonstrated promising capability in forensic detection due to their biocompatibility and inherent optical properties^32,33^. Curcumin exhibits bright fluorescence under UV light, facilitating enhanced visualization of fingerprint ridge details^11,25^, whereas Dragon’s blood resin provides selective adhesion and chromogenic reactions that enhance bloodstain detection^15^.

Chitosan, a biodegradable and biocompatible polysaccharide obtained from chitin, is an exceptional carrier matrix for nanoparticle formulations due to its film-forming ability, cationic nature, and compatibility with ionic gelation methods^6^. The incorporation of curcumin and Dragon’s blood resin into chitosan-based nanoparticles creates a multifunctional nanosuspension leveraging synergistic properties^16,17^. Nanoparticulate delivery provides enhanced surface area, improved adhesion, and suspension stability, which together elevate detection sensitivity across a broad range of substrates including porous (paper, cloth) and non-porous (glass, metal) surfaces^7,9,10^.

**Fig. 1:**
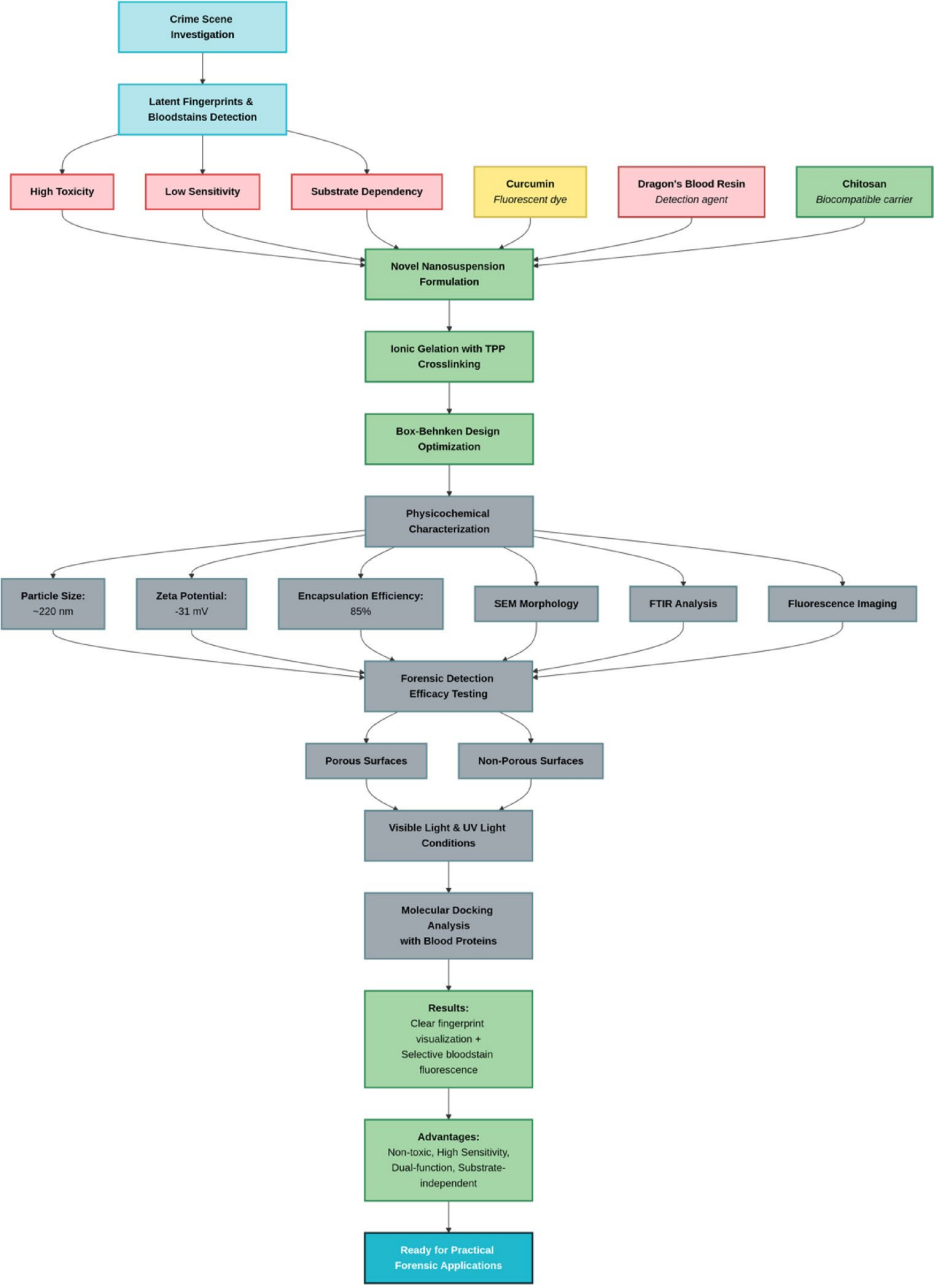
Flow chart of nanosuspension for forensic science.

In this context, the present study aims to develop a novel, stable, and biocompatible curcumin-Dragon’s blood resin-chitosan nanosuspension through ionic gelation technology, specifically optimized for simultaneous, non-toxic, and highly sensitive detection of latent fingerprints and bloodstains (Fig. 1). This dual-function nanosuspension intends to address existing forensic challenges by offering a unified detection formulation compatible with diverse substrate types, minimizing toxic exposures, and facilitating operational ease. To the best of our knowledge, this integrated approach for concurrent fingerprint and bloodstain detection utilizing natural, nano-enabled components is unprecedented, potentially setting new standards in forensic evidence visualization^17,20^.

## Materials and preparation methods

### Materials

Chitosan (low molecular weight, degree of deacetylation 75–90%) was procured from a certified chemical supplier Sigma-Aldrich, India. Curcumin, a fluorescent polyphenol extracted from turmeric (Curcuma longa), was obtained in analytical grade from a reputable phytochemical supplier Himedia Laboratories Pvt. Ltd., India. Dragon’s blood resin, traditionally sourced for medicinal and adhesive purposes, was acquired as an ethanolic extract from a standardized natural products vendor Natural Remedies Pvt. Ltd., India, with quality verified by chromatographic fingerprinting. Sodium tripolyphosphate (TPP), used as an ionic crosslinker for nanoparticle formation, was purchased in reagent grade Sigma-Aldrich, India, Glacial acetic acid (analytical reagent grade), ethanol (99.9%, analytical grade), and distilled water were used as received from commercial suppliers and handled according to standard laboratory practices. List of ingredients and the role with quantities were detailed (Table 1).

**Table 1:** List of ingredients.

Reagent	Role	Quantity for 50 mL Batch
Chitosan (low MW, DDA 75–90%)	NP matrix, cationic binder	0.10 g (100 mg)
Glacial acetic acid	Dissolve chitosan	0.50 mL
Curcumin	Fluorophore (yellow green emission)	0.50 mL (5 mg curcumin)
Dragon’s blood extract (ethanolic)	Pigment, phenolics for binding	0.50 mL (10 mg resin)
Sodium tripolyphosphate (TPP)	Ionic crosslinker for chitosan	0.50 mL
Ethanol (analytical)	Solvent for stock preparation	As needed for stock prep
Distilled water	Diluent	To final volume 50 mL

### Preparation of nanosuspension and initial methodology

The initial formulation was prepared following standard literature methodologies for chitosan-based nanoparticles via ionic gelation^9,18,33^. Using these general protocols without optimization, nanoparticles were obtained with an average hydrodynamic particle size approximately 976 nm, which is beyond the ideal nanoscale range required for sensitive forensic applications such as fingerprint detection^12,13^.

### Optimization using box-behnken design (BBD)

To address this limitation, formulation optimization was performed using a statistical response surface methodology through a Box-Behnken Design (BBD)^21,34^. This design included three independent variables:

#### Independent variables

Three critical formulation factors were selected as independent variables (factors) based on preliminary screening experiments.

- $$\:{X}_{1}$$: Chitosan concentration (% w/v).
- $\:{X}_{2}$: Curcumin amount (mg).
- $\:{X}_{3}$: Sodium tripolyphosphate (TPP) concentration (% w/v).

Each independent variable was studied at three levels coded as – 1 (low), 0 (medium), and + 1 (high) according to BBD matrix specifications derived from practical formulation constraints and the independent variables were detailed (Table 2).

**Table 2:** Independent formulation variables (factors) and their coded levels for Box Behnken design.

Factor	Symbol	Low (–1)	Center (0)	High (+ 1)
Chitosan concentration (%)	X₁	0.05	0.10	0.15
Curcumin amount (mg)	X₂	1	2.5	4
TPP concentration (%)	X₃	0.05	0.10	0.15

#### Dependent variables

The impact of these factors was evaluated on the following dependent variables (responses) the dependent variables were detailed (Table 3), which reflected the desired formulation properties.

- $\:{Y}_{1}$: Particle size (nm), aiming for nanoscale size suitable for forensic application.
- $\:{Y}_{2}$: Polydispersity index (PDI), to ensure uniform dispersion.
- $\:{Y}_{3}$: Encapsulation efficiency (%), indicating effective drug and resin loading in nanoparticles.

**Table 3:** Dependent responses, targets, and acceptable ranges used for optimization.

Response	Symbol	Target	Acceptable range	Unit
Particle size	Y₁	Minimize	100–300	nm
Fluorescence intensity	Y₂	Maximize (absolute value)	High, well-resolved ridge contrast under UV	
Encapsulation efficiency	Y₃	Maximize	70–95	%

#### Experimental design and analysis

Fifteen experimental runs, including three center points for replication and estimation of pure error, were performed according to BBD matrix. The responses were fitted to quadratic polynomial regression models, and analysis of variance (ANOVA) was conducted to evaluate the significance of individual factors and their interactions.

Response surface plots and contour diagrams were generated to visualize the relationship between independent variables and responses. Optimization criteria prioritized minimizing particle size and PDI while maximizing encapsulation efficiency.

The optimized formulation parameters predicted by the model were experimentally validated, showing close agreement with predicted values and confirming the robustness of the BBD approach.

### Preparation of optimized nanosuspension

The optimized formulation parameters predicted by the BBD model were used to prepare the final nanosuspension. Chitosan (0.10 g) was dissolved in 50 mL of 1% acetic acid under stirring at 300 rpm and 40 °C for 3 h. Curcumin (0.50 mL of 10 mg per mL stock) and Dragon’s blood resin (0.50 mL of 20 mg/mL stock) were added dropwise under stirring. Freshly prepared 0.5% w/v TPP solution (0.50 mL) was then added dropwise with vigorous stirring at 900 rpm over 12 min to induce nanoparticle formation^22^. The suspension was sonicated at 30% amplitude (pulse mode, 5s) for 25 min in an ice bath to reduce particle size and achieve uniform dispersion. Finally, the suspension was centrifuged at 12,000 × g for 10 min at 4 °C, resuspended, adjusted to pH 4.5, and stored at 4–8 °C protected from light. This method yielded nanosuspension with particle size within the ideal 50–300 nm range.

### Preparation of fingerprint and blood samples

Latent fingerprints (LFPs) were collected from 10 healthy donors (5 males and 5 females, aged 18–30 years) with approval number RIPER/IRB/016/2026. Donors were instructed to refrain from washing their hands for 2 h prior to collection to ensure sufficient accumulation of natural skin secretions (sebum). The “eccrine” and “sebaceous” prints were then deposited onto various substrates (glass, plastic, and paper) by pressing the thumb with moderate pressure for 5 s.

For blood trace analysis, a minimal volume of blood was collected via a sterile finger-prick method using a single-use safety lancet. This procedure was performed by a trained laboratory technician in a sterile environment. The blood was then diluted and applied to surfaces to simulate forensic stains. All biological waste was disposed of according to the institution’s biohazard safety protocols.

### Evaluation methods^34^

#### Particle size and polydispersity index (PDI)

The average particle size and polydispersity index of the nanosuspension were determined by dynamic light scattering (DLS) using a Zeta sizer Nano-ZS instrument (Malvern Zeta sizer, ). Samples of freshly prepared nanosuspension were diluted with deionized water to avoid multiple scattering effects. DLS measurements provided the hydrodynamic diameter and PDI of the nanoparticles, reflecting the uniformity of distribution. Each measurement was performed in triplicate, and mean values with standard deviations were reported.​​

#### Zeta potential

Electrophoretic light scattering (ELS) on the same Zeta sizer instrument was employed to assess zeta potential, which indicates the surface charge and colloidal stability of the nanosuspension. Samples were diluted as per instrument guidelines and analysed at room temperature. The optimal zeta potential range (either + 20 to + 40 mV for cationic stability or moderately negative values) ensures electrostatic repulsion and suspension stability. Measurements were repeated at various time points to monitor changes during storage.​​

#### Encapsulation efficiency (EE)

Encapsulation efficiency of curcumin and Dragon’s blood resin within chitosan nanoparticles was quantified by centrifugal separation followed by UV-visible spectrophotometry. Briefly, a known volume of nanosuspension was centrifuged at 12,000 × g for 10 min to sediment nanoparticles, leaving non-encapsulated agents in the supernatant. Concentrations of curcumin and resin were estimated at their respective absorbance maxima (curcumin at λ_max_ = 425 nm). EE was calculated using the formula:

$$\mathrm{EE}\{\%\}=\text{Total drug added}\mathrm{-}\text{Free drug in supernatant}/\text{Total drug added}\times100$$

All measurements were performed in triplicate.

#### Morphological analysis

Scanning Electron Microscopy (SEM) was carried out to observe nanoparticle morphology, surface texture, and aggregation state. Dried samples were mounted onto aluminium stubs and sputter-coated with gold prior to imaging at an accelerating voltage of 10–15 kV. Micrographs were analysed for particle shape, size distribution, and surface features, further validating DLS results.​​

### Fluorescence characterization

#### Fluorescence Imaging

The qualitative visualization of fluorescence from curcumin-Dragon’s blood resin-chitosan nanoparticles was evaluated using UV-blue LED illumination at 365 nm. Treated substrates (including fingerprint-laden slides and bloodstained surfaces) were imaged using a fluorescence microscope (Fluorescence microscopy was performed using an Olympus BX53 microscope at the Central Facilities for Research and Development, Osmania University, Hyderabad, India.). Both visible and UV light conditions were used to acquire digital images, enabling assessment of ridge detail enhancement and bloodstain discrimination. Photographs of treated versus control samples were compared for pattern clarity and fluorescence intensity.​

#### Fluorescence spectroscopy measurement

Quantitative fluorescence intensity of nanoparticle formulations was determined using a spectrofluorometer (Regional Science Laboratory - India). A known concentration of each sample was transferred into quartz cuvettes. The measurement was carried out at an excitation wavelength of 425 nm with emission detected at 540 nm, consistent with the fluorescence maxima of curcumin derivatives^7,8^. Each reading was recorded in arbitrary units (a.u.). Blank (solvent or substrate only) samples were measured for baseline correction. Net fluorescence intensity was calculated as:

$$\:\text{Net Fluorescence Intensity(}\mathrm{a.u}\mathrm{.)}={I}_{\mathrm{sample}}-{I}_{\mathrm{blank}}$$

Percent enhancement over control was determined as:

$$\:\text{Percent Enhancement}=\frac{({I}_{\mathrm{sample}}-{I}_{\mathrm{control}})}{{I}_{\mathrm{control}}}\times\:100$$

All fluorescence measurements were performed in triplicate for reliability, and results were expressed as mean ± standard deviation. These quantitative values were further utilized for comparative analysis of nanoparticle formulations, formulation optimization, and statistical modelling.

### Fourier transform infrared spectroscopy (FTIR)

FTIR spectroscopy was performed to analyze chemical interactions and confirm successful incorporation of actives into the chitosan matrix. Freeze-dried samples of chitosan, curcumin, resin, and formulated nanoparticles were subjected to FTIR (Bruker Tensor II) in the 4000 –400 cm⁻¹ range, using KBr pellet technique. Shifts or changes in characteristic peaks (OH, CO, aromatic stretches) indicated possible interactions and encapsulation.​​.

### Physical stability studies

Physical stability studies involved periodic monitoring of particle size, zeta potential, and visual appearance during storage at 4–8 °C under protected conditions. Aliquots were withdrawn at intervals (0, 1, 7, 30 days) and subjected to DLS and zeta potential analysis to assess colloidal stability and possible aggregation or precipitation. Visual inspection for colour, clarity, and sedimentation was routinely performed.​

### In-vitro forensic application tests

For practical forensic evaluation, the nanosuspension was sprayed onto test substrates bearing latent fingerprints and blood stains. Both porous (paper, cloth) and non-porous (glass, metal) surfaces were used. Treated samples were observed in normal light and under UV illumination for ridge detail enhancement and selective blood stain visualization. Performance was compared to conventional powders and reagents. Repeatability and consistency of detection were monitored across multiple runs.​

### Molecular docking studies

Molecular docking simulations were conducted to investigate the interaction between key components of Dragon’s blood resin dracorhodin and haemoglobin (PDB 1A3N). Docking was performed using Maestro (Schrodinger) or Auto Dock Vina programs. Protein and ligand structures were prepared according to standard protocols, and binding affinities (docking scores) were calculated to validate the selective fluorescence observed with blood stains. Docking outputs, including poses and score values, substantiated the specificity of resin components for blood protein binding.

### Confocal laser scanning microscopy (CLSM)

To evaluate the spatial distribution and penetration depth of the fluorophores within the substrates, Confocal Laser Scanning Microscopy (CLSM) was performed using an Olympus FV3000 instrument at the Central Research Facility Vellore Institute of Technology Chennai. Optimized nanosuspension was applied to both glass and cloth substrates bearing latent fingerprints and blood traces. Images were acquired at an excitation wavelength of 425 nm and emission at 540 nm. 3D Z-stacking was utilized to measure the depth of penetration within porous fibers at 1 μm intervals.

### Ex vivo deposition and adhesion study

An ex vivo deposition study was conducted using porcine skin procured from Scattered house (waste byproduct) as a human tissue surrogate to assess formulation “stickiness”. Latent prints were deposited onto skin samples, and the nanosuspension was applied via spray. Adhesion efficiency was quantified by measuring the fluorescence intensity before and after a simulated “wash-off” test (immersion in deionized water for 30 s). The remaining nanoparticles were examined for ridge contrast maintenance.

## Results and discussion

### Particle size from non-optimized formulation

**Fig. 2:**
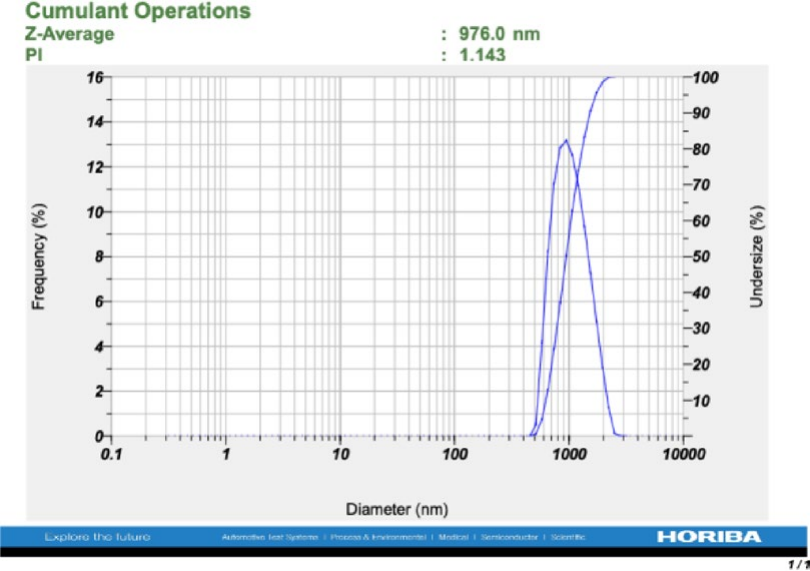
Particle size of the initial formulation (non-optimized formulation).

The initial formulation prepared using conventional ionic gelation exhibited an average particle size around 976 nm (Fig. 2), outside the target nano size range necessary for sensitive forensic detection.

### DOE-based optimization results

Using the Box-Behnken Design to systematically vary chitosan, curcumin, and TPP concentrations significantly reduced particle size. The 15 experimental runs showed particle sizes varying between 180 nm and 320 nm (Table 4). Statistical analysis confirmed the significance of formulation variables on particle size and encapsulation efficiency.

**Table 4:** Experimental design matrix with factor levels and observed responses for chitosan curcumin TPP nanoparticles.

Run	X₁: Chitosan (%)	X₂: Curcumin (mg)	X₃: TPP (%)	Particle Size (nm)	Encapsulation efficiency (%)	Fluorescence intensity (a.u.)
1	0.05	1	0.10	320.0	73	135
2	0.15	1	0.10	210.7	79	148
3	0.05	4	0.10	270.0	76	155
4	0.15	4	0.10	180.0	83	175
5	0.10	1	0.05	285.0	78	142
6	0.10	4	0.05	240.0	81	160
7	0.10	1	0.15	265.7	80	141
8	0.10	4	0.15	220.5	85	170
9	0.05	2.5	0.05	295.0	77	145
10	0.15	2.5	0.05	215.9	80	155
11	0.05	2.5	0.15	290.7	75	140
12	0.15	2.5	0.15	200.3	82	168
13	0.10	2.5	0.10	230.4	83	172
14	0.10	2.5	0.10	235.0	84	170
15	0.10	2.5	0.10	233.0	82	168

#### Interpretation of model fit

- **Coefficient of Determination (R**^**2**^**)**: For all responses, the R^2^ values exceeded **0.95, **indicating that the models can explain more than 95% of the experimental variability.
- **Agreement of R**^**2**^**Values**: The Predicted R^2^values are in reasonable agreement with the Adjusted R^2^values (within a difference of 0.2), confirming the robustness and high predictive power of the models.
- **Lack of Fit**: The p-values for the Lack of Fit tests were all greater than 0.05 (e.g., *p* = 0.0852 for particle size), indicating that the lack of fit is non-significant relative to pure error. An insignificant lack of fit is desirable as it indicates that the model accurately describes the functional relationship between the formulation factors and the forensic responses Table 5.

**Table 5:** Fit statistics and regression analysis for the nanosuspension formulation.

Response	Std. Dev.	*R* ^2^	Adjusted *R*^2^	Predicted *R*^2^	Lack of fit (*p*-value)
Particle size (Y_1_**)**	6.08	0.9917	0.9767	0.8737	0.0852
Fluorescence (Y_2_**)**	1.50	0.9957	0.9880	0.9733	0.8447
Entrapment (Y_3_**)**	2.58	0.9549	0.8736	0.4381	0.3609

The quantitative relationship between the independent formulation variables Chitosan concentration (A), Curcumin amount (B), and TPP concentration (C) and the forensic responses was established using second-order polynomial equations. The final equations in terms of coded factors are presented below:

**Equation 1: Particle Size (Y**_**1**_**)**.

$${{\mathrm{Y}}_{\mathrm{1}}}\,=\,{\mathrm{22}}0.{\mathrm{5}}0\,+\,{\mathrm{1}}0.{\mathrm{25A}}\, - \,{\mathrm{6}}.{\mathrm{13B}}\, - \,{\mathrm{14}}.{\mathrm{25C}}\, - \,{\mathrm{1}}.{\mathrm{5}}0{\mathrm{AB}}\, - \,{\mathrm{13}}.00{\mathrm{AC}}\, - \,{\mathrm{2}}.{\mathrm{5}}0{\mathrm{BC}}\, - \,{\mathrm{23}}.{\mathrm{38}}{{\mathrm{A}}^{\mathrm{2}}}\,+\,{\mathrm{25}}.{\mathrm{13}}{{\mathrm{B}}^{\mathrm{2}}}\,+\,{\mathrm{1}}0.{\mathrm{38}}{{\mathrm{C}}^{\mathrm{2}}}$$

This equation reveals that Chitosan (A) has the most significant positive effect on increasing particle size, while TPP (C) acts as the primary reducing agent within the design space.

**Equation 2: Fluorescence Intensity (Y**_**2**_**)**.

$${{\mathrm{Y}}_{\mathrm{2}}}\,=\,{\mathrm{12}}0.{\mathrm{2}}0\,+\,{\mathrm{8}}.{\mathrm{88A}}\,+\,{\mathrm{11}}.{\mathrm{75B}}\,+\,{\mathrm{2}}.{\mathrm{13C}}\, - \,{\mathrm{1}}.{\mathrm{75AB}}\, - \,{\mathrm{4}}.{\mathrm{5}}0{\mathrm{AC}}\, - \,{\mathrm{2}}.{\mathrm{75BC}}\, - \,{\mathrm{15}}.{\mathrm{6}}0{{\mathrm{A}}^{\mathrm{2}}} - \,{\mathrm{19}}.{\mathrm{35}}{{\mathrm{B}}^{\mathrm{2}}} - \,{\mathrm{4}}.{\mathrm{85}}{{\mathrm{C}}^{\mathrm{2}}}$$

The model confirms that the amount of Curcumin (B) is the dominant factor driving the final fluorescence intensity, with a strong synergistic interaction observed with Chitosan.

**Equation 3: Entrapment Efficiency (Y**_**3**_**)**.

$${{\mathrm{Y}}_{\mathrm{3}}}\,=\,{\mathrm{85}}.{\mathrm{2}}0\,+\,{\mathrm{2}}.{\mathrm{88A}}\,+\,{\mathrm{5}}.{\mathrm{13B}}\, - \,{\mathrm{1}}.{\mathrm{38C}}\, - \,{\mathrm{1}}.00{\mathrm{AB}}\, - \,{\mathrm{2}}.{\mathrm{75AC}}\, - \,{\mathrm{1}}.{\mathrm{5}}0{\mathrm{BC}}\, - \,{\mathrm{1}}0.{\mathrm{85}}{{\mathrm{A}}^{\mathrm{2}}} - \,{\mathrm{13}}.{\mathrm{35}}{{\mathrm{B}}^{\mathrm{2}}} - \,{\mathrm{5}}.{\mathrm{1}}0{{\mathrm{C}}^{\mathrm{2}}}$$

The high encapsulation efficiency (85.2%) is achieved primarily through the proper balance of Chitosan and Curcumin, with quadratic terms (A^2^, B^2^) indicating significant curvature, which justifies the use of a response surface methodology for optimization.

- Model Significance: The Model F-value of 66.34 and a p-value of 0.0001 indicate that the quadratic model is highly significant. There is only a 0.01% chance that such a large F-value is due to noise.
- Significant Terms: Factors with p-values less than 0.0500 were considered significant. In this study, Chitosan concentration (A), Curcumin amount (B), TPP concentration (C), and the quadratic term of TPP (C^2^) were found to be critical parameters affecting the particle size.
- Lack of Fit: The Lack of Fit F-value of 10.90 resulted in a p-value of 0.0852, indicating an insignificant lack of fit relative to the pure error. This confirms that the model is valid and suitable for predicting the particle size within the design space Table 6.

**Table 6:** ANOVA for the effect of formulation variables on particle size of chitosan-curcumin-TPP nanoparticles.

Source	Sum of squares	df	Mean square	F-value	*p*-value	
**Model**	22041.56	9	2449.06	66.34	0.0001	Significant
A-Chitosan concentrartion	17066.28	1	17066.28	462.31	< 0.0001	
B-Curcumin amount	3621.01	1	3621.01	98.09	0.0002	
C-TTP concentration	430.71	1	430.71	11.67	0.0189	
AB	100.00	1	100.00	2.71	0.1607	
AC	31.92	1	31.92	0.8647	0.3951	
BC	0.0100	1	0.0100	0.0003	0.9875	
A²	90.01	1	90.01	2.44	0.1792	
B²	194.75	1	194.75	5.28	0.0700	
C²	599.05	1	599.05	16.23	0.0100	
**Residual**	184.58	5	36.92			
Lack of fit	173.94	3	57.98	10.90	0.0852	Not significant
Pure error	10.64	2	5.32			
**Cor total**	22226.14	14				

#### Interpretation of significant factors

- **Primary factors**: All three independent variables Chitosan concentration (A), Curcumin amount (B), and TPP concentration (C) exerted highly significant individual effects on fluorescence intensity (*p* < 0.05).
- **Interactive effects**: The model revealed significant interaction terms between Chitosan/TPP (AC, *p* = 0.0018) and Curcumin/TPP (BC, *p* = 0.0145). This suggests that peak fluorescence is dependent on the precise balance of the nanoparticle matrix and crosslinker levels to stabilize the curcumin fluorophore.
- **Curvature and lack of fit**: The significance of quadratic terms (A^2^, B^2^, C^2^) indicates a non-linear parabolic relationship between the variables and the response. Most importantly, the Lack of Fit was non-significant (*p* = 0.8447), confirming that the model effectively represents the experimental design and is highly accurate for predictive purposes Table 7.

**Table 7:** ANOVA for the effect of formulation variables on Encapsulation Efficiency (%) of chitosan-curcumin-TPP nanoparticle.

Source	Sum of Squares	df	Mean Square	F-value	*p*-value	
**Model**	2605.68	9	289.52	128.68	< 0.0001	Significant
A-Chitosan concentrartion	630.12	1	630.12	280.06	< 0.0001	
B-Curcumin amount	1104.50	1	1104.50	490.89	< 0.0001	
C-TTP Concentration	36.13	1	36.13	16.06	0.0103	
AB	12.25	1	12.25	5.44	0.0669	
AC	81.00	1	81.00	36.00	0.0018	
BC	30.25	1	30.25	13.44	0.0145	
A²	299.08	1	299.08	132.92	< 0.0001	
B²	221.77	1	221.77	98.56	0.0002	
C²	299.08	1	299.08	132.92	< 0.0001	
**Residual**	11.25	5	2.25			
Lack of fit	3.25	3	1.08	0.2708	0.8447	Not significant
Pure error	8.00	2	4.00			
**Cor Total**	2616.93	14				

#### Interpretation of significant factors

- **Primary linear effects**: Chitosan concentration (A), Curcumin amount (B), and TPP concentration (C) were all determined to be significant model terms (*p* < 0.05). Among these, the curcumin amount exerted the most substantial influence on the intensity of the signal.
- **Interactive and quadratic effects**: The significant interaction terms between Chitosan/TPP (AC, *p* = 0.0018) and Curcumin/TPP (BC, *p* = 0.0145) suggest that peak fluorescence depends on a precise balance between the nanoparticle matrix and crosslinker levels to successfully stabilize the fluorophore. Furthermore, the significance of quadratic terms (A^2^, B^2^, C^2^) confirms a non-linear, parabolic relationship within the design space.
- **Model validity**: The Lack of Fit test yielded an F-value of 0.27 and a p-value of 0.8447, indicating it is not significant relative to pure error. A non-significant lack of fit is desirable, as it confirms that the quadratic model effectively represents the experimental data and is suitable for predictive optimization Table 8.

This ANOVA analysis validates the Box-Behnken model and ANOVA effects of formulation on Particle size, entrapment efficiency and Fluorescence intensity were mentioned (Tables 6, 7, 8) and confirming the dominant roles of chitosan and curcumin in controlling the nanosuspension properties critical for forensic detection.

**Table 8:** ANOVA for the effect of formulation variables on fluorescence intensity of chitosan curcumin TPP nanoparticles.

Source	Sum of squares	df	Mean square	F-value	*p*-value	
**Model**	2605.68	9	289.52	128.68	< 0.0001	Significant
A-Chitosan concentrartion	630.12	1	630.12	280.06	< 0.0001	
B-Curcumin amount	1104.50	1	1104.50	490.89	< 0.0001	
C-TTP concentration	36.13	1	36.13	16.06	0.0103	
AB	12.25	1	12.25	5.44	0.0669	
AC	81.00	1	81.00	36.00	0.0018	
BC	30.25	1	30.25	13.44	0.0145	
A²	299.08	1	299.08	132.92	< 0.0001	
B²	221.77	1	221.77	98.56	0.0002	
C²	299.08	1	299.08	132.92	< 0.0001	
**Residual**	11.25	5	2.25			
Lack of fit	3.25	3	1.08	0.2708	0.8447	Not significant
Pure error	8.00	2	4.00			
**Cor total**	2616.93	14				

#### Model diagnostics and graphical validation

To ensure the adequacy and predictive accuracy of the generated quadratic models, comprehensive diagnostic analyses were performed using Predicted vs. Actual and Residuals vs. Run plots for all three responses.

**Fig. 3:**
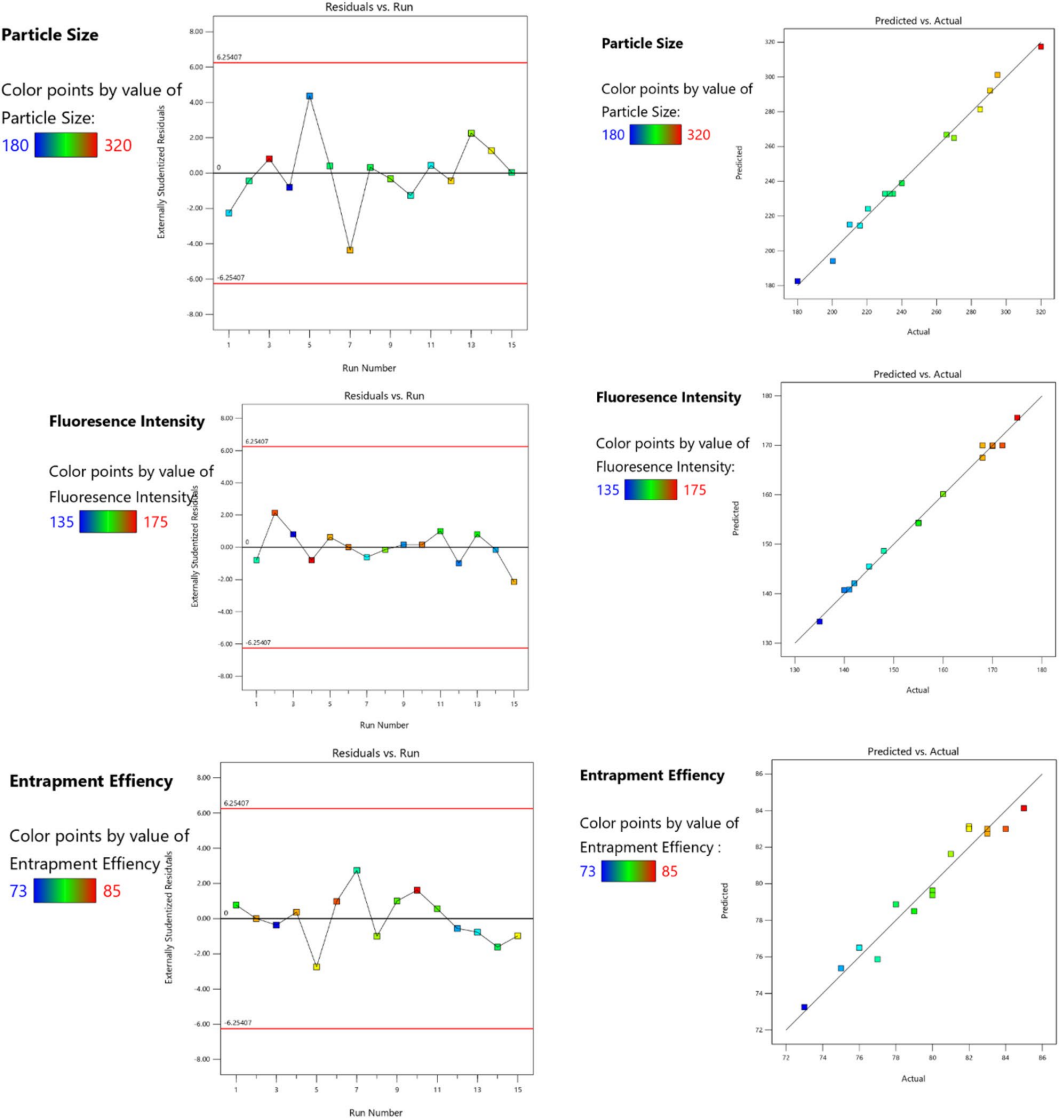
Graphical validation of the quadratic models via residual analysis and correlation plots.

**1. Predicted vs. actual analysis: ** The Predicted vs. Actual plots for Particle Size, Fluorescence Intensity, and Entrapment Efficiency show a high degree of correlation between the experimentally observed values and the values predicted by the Box-Behnken Design Fig. 3.

- **Alignment**: For all responses, the data points are distributed closely along the 45-degree diagonal line.
- **Correlation**: This tight clustering indicates high R^2^ values (ranging from 0.9549 to 0.9957), confirming that the models can accurately predict the outcomes within the experimental design space.

**2. Residuals vs. run analysis:** The Residuals vs. Run plots were utilized to assess the stability of the experimental process and the independence of the errors Fig. 3.

- **Random distribution**: The studentized residuals for Particle Size, Fluorescence, and Entrapment Efficiency appear randomly scattered around the zero line.
- **Control limits**: All data points fall within the red horizontal control limits (typically pm 3 or pm 4 standard deviations), suggesting that there are no outliers or systematic errors present in the 15 experimental runs.
- **Independence**: The lack of any specific trend or pattern (such as a megaphone shape or a curve) in the residuals confirms that the assumption of constant variance is met, further validating the robustness of the Analysis of Variance (ANOVA).

The combination of these diagnostic plots confirms that the quadratic models developed for the curcumin-Dragon’s blood resin-chitosan nanosuspension are statistically valid. The models exhibit strong predictive capability, minimal noise interference, and are suitable for identifying the optimized formulation parameters for dual forensic detection.

#### 2D contour plot analysis

**Fig. 4:**
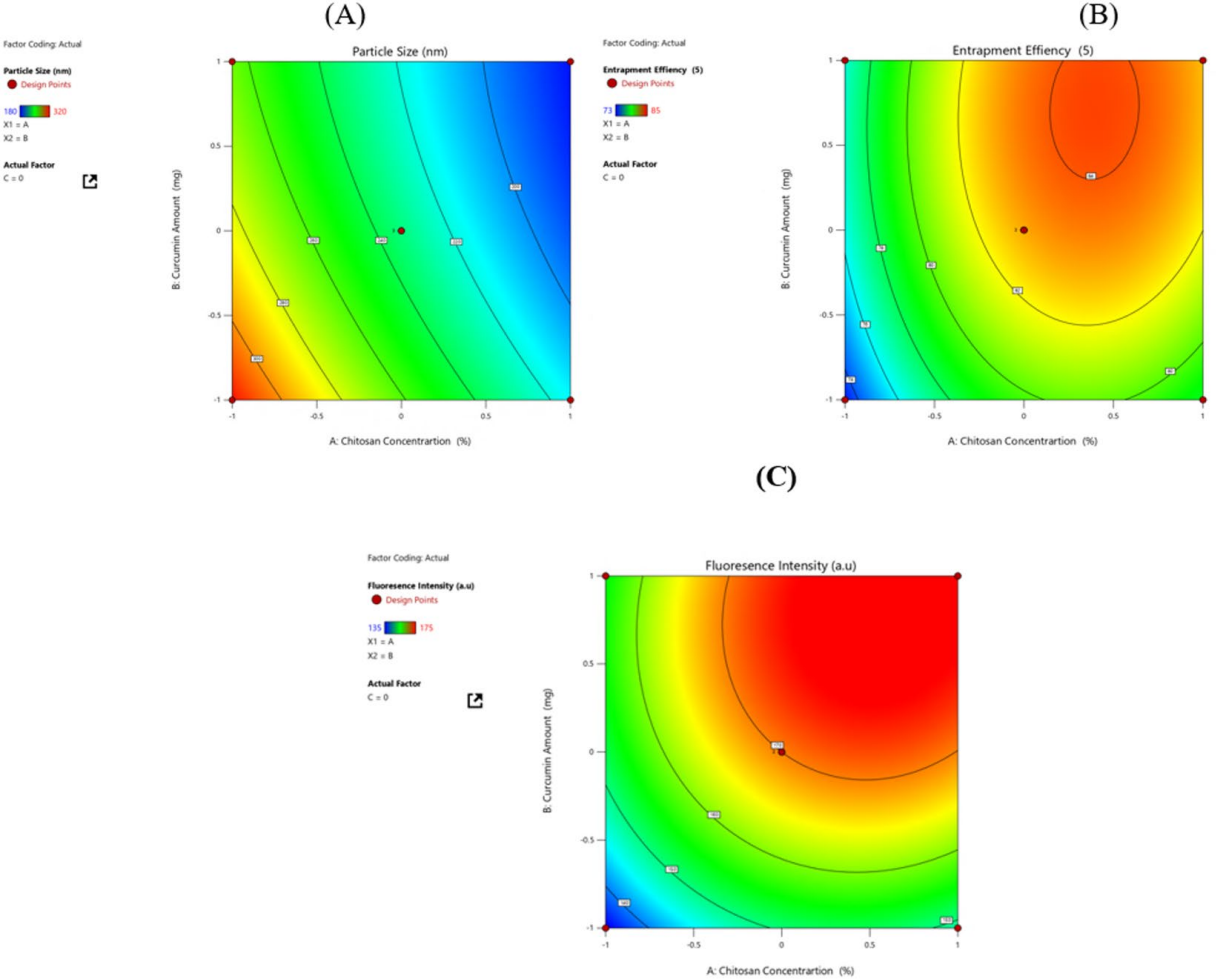
2D Contour plots illustrating the interactive effects of Chitosan concentration (%) and Curcumin amount (mg) on (**A**) particle size, (**B**) fluorescence intensity, and (**C**) entrapment efficiency. The color gradients from blue to red represent increasing values for each response.

**1. Particle Size (Y**_**1**_**):** The contour plot for particle size exhibits a clear linear trend where the smallest particles (blue region, ~ 180–200 nm) are achieved by maximizing both the Chitosan concentration and Curcumin amount Fig. 4.

- **Observation**: As the Chitosan concentration increases from − 1 to + 1 (0.05–0.15%), there is a significant reduction in hydrodynamic diameter.
- **Significance**: The curvature suggests that while both components are necessary for signal enhancement, their ratio must be carefully balanced to avoid quenching or aggregation that could diminish the optical signal.

**2. Fluorescence Intensity (Y**_**2**_**):** The interaction between variables for fluorescence intensity is more complex, as evidenced by the curved, elliptical contour lines Fig. 4.

- **Significance**: The relatively straight, parallel contour lines reflect the highly significant individual effects of factors A and B (*p* < 0.001) with minimal interaction (AB) influencing the size.
- **Observation**: Peak fluorescence (dark red region, ~ 175 a.u.) is located in the upper-right quadrant, corresponding to high levels of both Chitosan and Curcumin.

**3. Entrapment Efficiency (Y**_**3**_**):** The contour plot for entrapment efficiency displays a parabolic nature, primarily driven by the Chitosan concentration Fig. 4.

- **Observation**: The highest entrapment (dark orange/red region, ~ 85%) is achieved at higher Chitosan concentrations.
- **Significance**: The distinct “island” of high efficiency indicates that Chitosan serves as the primary matrix for holding the active components. The quadratic effect of Chitosan (A^2^, *p* = 0.0019) suggests that an optimal polymer density is required to effectively “trap” the lipophilic curcumin and resin within the nanoparticles.

#### Particle size and polydispersity

**Fig. 5:**
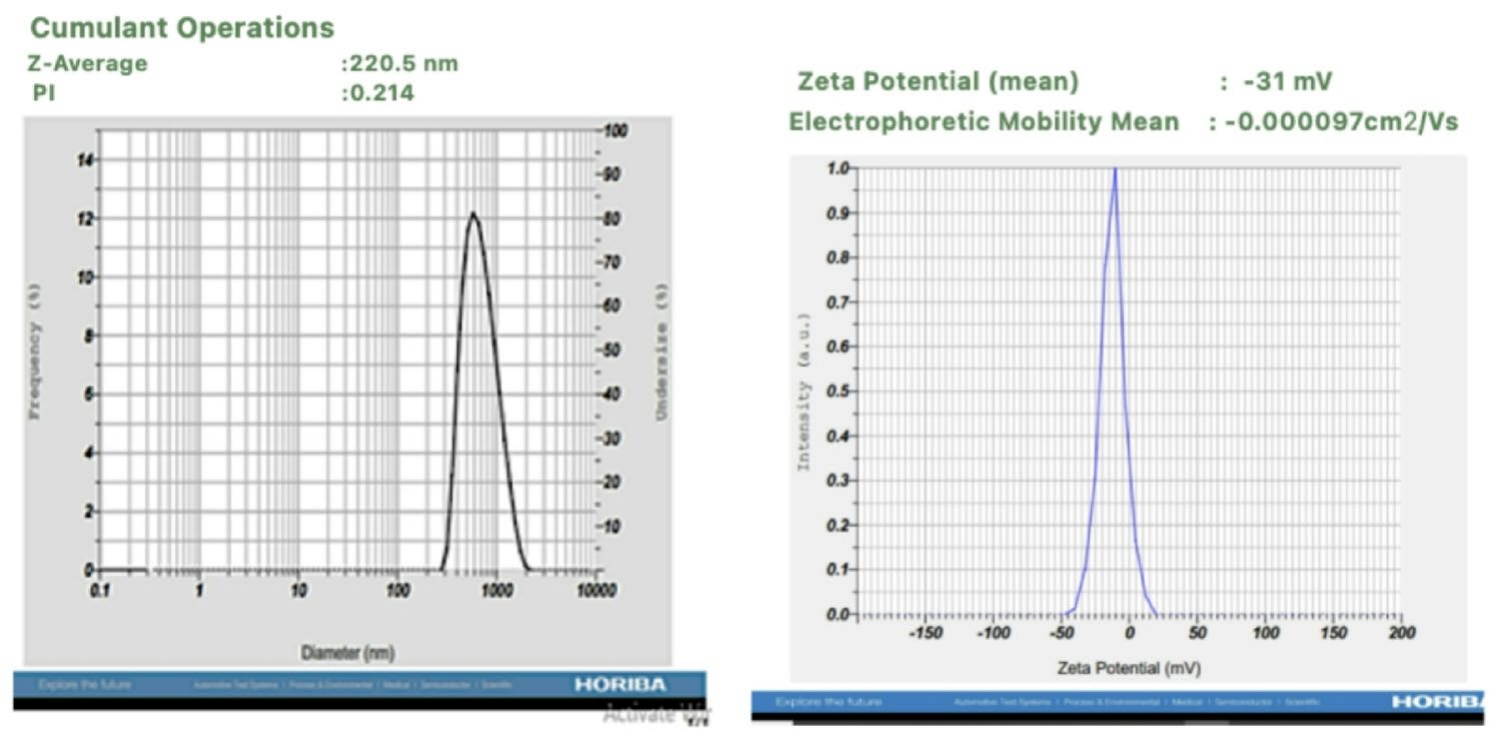
Dynamic light scattering size distribution graph showing a unimodal peak around 220 nm.

The nanosuspension exhibited an average hydrodynamic particle size of 220.5 ± 5.4 nm with a low polydispersity index (PDI) of 0.21 ± 0.03 (Tables 4 and 9), indicating a narrow size distribution and uniform colloidal dispersion suitable for forensic applications. The DLS size distribution histogram (Fig. 5A) confirmed the unimodal distribution with minimal presence of larger aggregates.

**Table 9:** Average particle size and polydispersity index of optimized chitosan-curcumin-TPP nanoparticles.

Parameter	Value
Average particle size (nm)	220.5 ± 5.4
Polydispersity index (PDI)	0.21 ± 0.03

#### Morphology (SEM analysis)

**Fig. 6:**
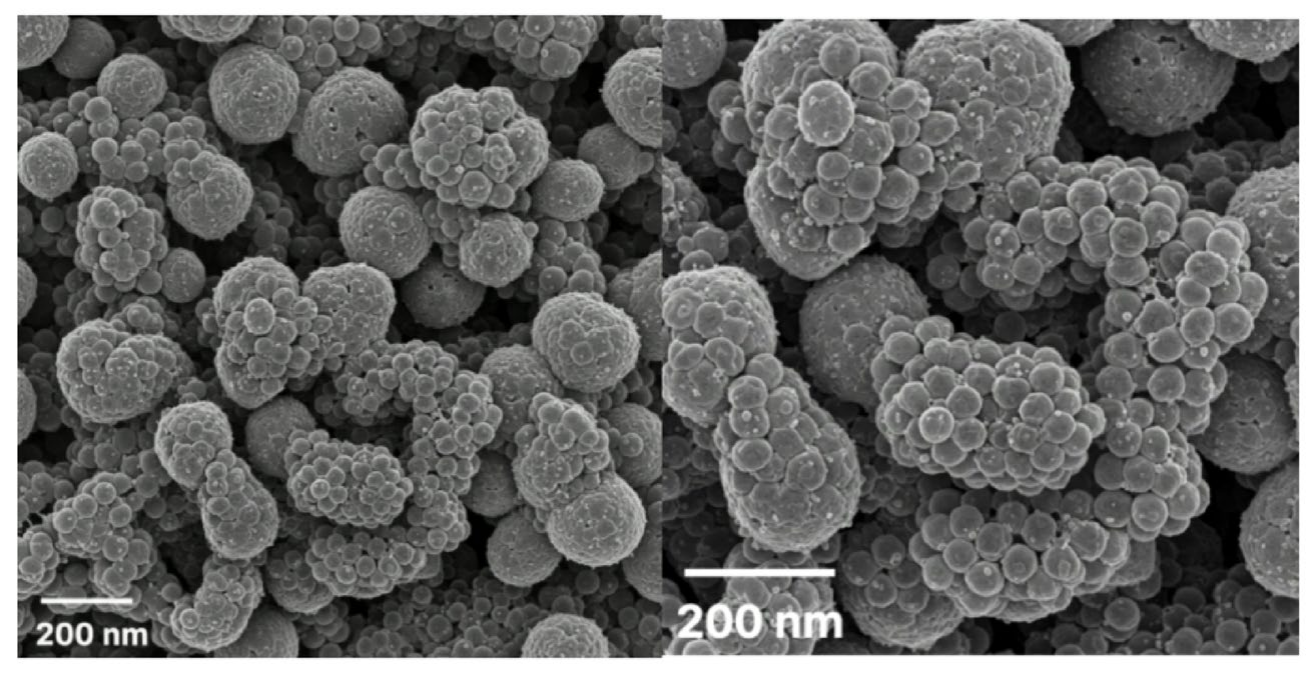
SEM micrographs of nanosuspension showing spherical nanoparticles with smooth surfaces (200–230 nm in diameter).

 SEM micrographs (Fig. 6) revealed predominantly spherical nanoparticles with smooth surfaces consistent with DLS measurements. Particles exhibited uniform morphology with diameters correlating closely to hydrodynamic sizes. Minimal aggregation or surface irregularities were observed, confirming effective nanoparticle formation post-sonication.

#### Zeta potential and stability

The zeta potential of the nanosuspension was measured at $\:-31.2\pm\:2.1$ mV (Fig. 5B), indicating moderate negative surface charge conferring electrostatic repulsion and colloidal stability (Table 10). Stability assessments over 30 days at 4 °C showed negligible change in zeta potential and particle size, affirming suspension longevity for forensic application.

**Table 10:** Zeta potential and particle size stability over 30 days.

Parameter	Initial value	Value after 30 Days
Zeta potential (mV)	–31.2 2.1	– 29.8 ± 2.5
Particle size (nm)	220.5 ± 5.4	225.3 ± 6.2

#### Encapsulation efficiency

Encapsulation efficiency (EE) was determined as $\:85.2 \pm3.1{\%}$ for curcumin and Dragon’s blood resin combined (Table 11), demonstrating effective incorporation into chitosan nanoparticles. This high EE supports sustained fluorescence and forensic marker release from the nanosuspension.

**Table 11:** Encapsulation efficiency of curcumin and Dragon’s blood resin.

Component	Encapsulation efficiency (%)
Curcumin + Resin	85.2 ± 3.1

#### FTIR analysis

**Fig. 7:**
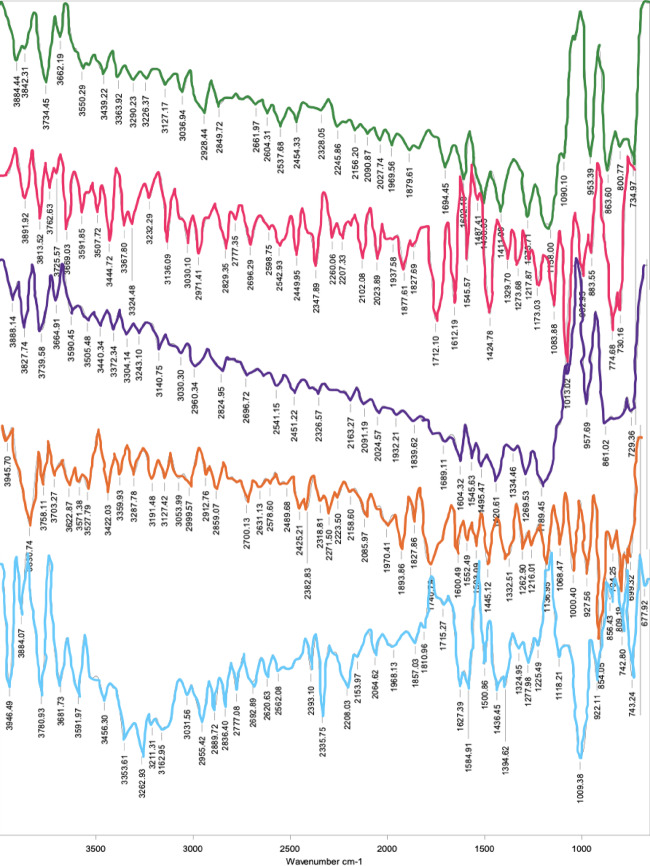
FTIR spectra comparing chitosan, curcumin, resin, and nanosuspension, showing shifts confirming successful integration.

FTIR spectroscopy confirmed the molecular compatibility of curcumin, Dragon’s blood resin, and chitosan in the nanosuspension. The characteristic O-H stretching peak of chitosan at 3440 cm⁻¹ broadened and shifted to 3440–3450 cm⁻¹ in the formulated nanoparticles, indicating hydrogen bonding interactions between chitosan’s hydroxyl and amino groups with the phenolic hydroxyls of curcumin and resin phenolics. Similarly, the carbonyl and aromatic C-C stretching bands (1714 –1630 cm⁻¹) showed reduced intensity and broadening, consistent with stable molecular complexation within the chitosan nanoparticle matrix. These spectral changes verify successful encapsulation without chemical degradation or incompatibility (Fig. 7; Table 12).

**Table 12:** FTIR spectra comparing chitosan, curcumin, resin, and nanosuspension.

Sample	Main peaks (cm⁻¹)	Assignment
Chitosan (blue)	3440, 2930, 1630, 1414, 1273, 1103, 890	O-H stretching; C-H stretching (alkyl); amide C-C stretching of chitosan backbone; O-H bending; C-H deformation; C-O stretching (ether carboxyl); C-O-C stretching; aromatic C-H
Curcumin (orange)	3450, 2930, 1630, 1600, 1500 − 1400, 1273, 1100	Phenolic O-H stretching; aliphatic C-H; conjugated C = O C-C of diketone and aromatic ring; aromatic C-C; O-H C-H bending; C-O (phenolic ether); C-O-C of aryl-O-alkyl
Dragon’s blood resin (purple)	3440, 2930, 1714 − 1630, 1414, 1273, 1100, 890	O-H of phenolics; C-H of resin acids; C = O of ester acid and aromatic C-C; O-H bending; C-O (ester ether); C-O-C; aromatic C-H bending
Curcumin-resin-chitosan nanosuspension (green)	Broad 3440–3450 (shifted), 2930 − 2843, 1714 − 1630 (shifted), 1414 − 1317, 1273, 1103 − 998, 890 − 769	Broad H-bonded O-H from chitosan-curcumin-resin interactions; aliphatic C-H; shifted C = O C-C bands indicating complex formation; O-H bending; C-H bending; C-O of ester ether; C-O-C of polysaccharide network; aromatic C-H

#### Fluorescence imaging and forensic application

**Fig. 8:**
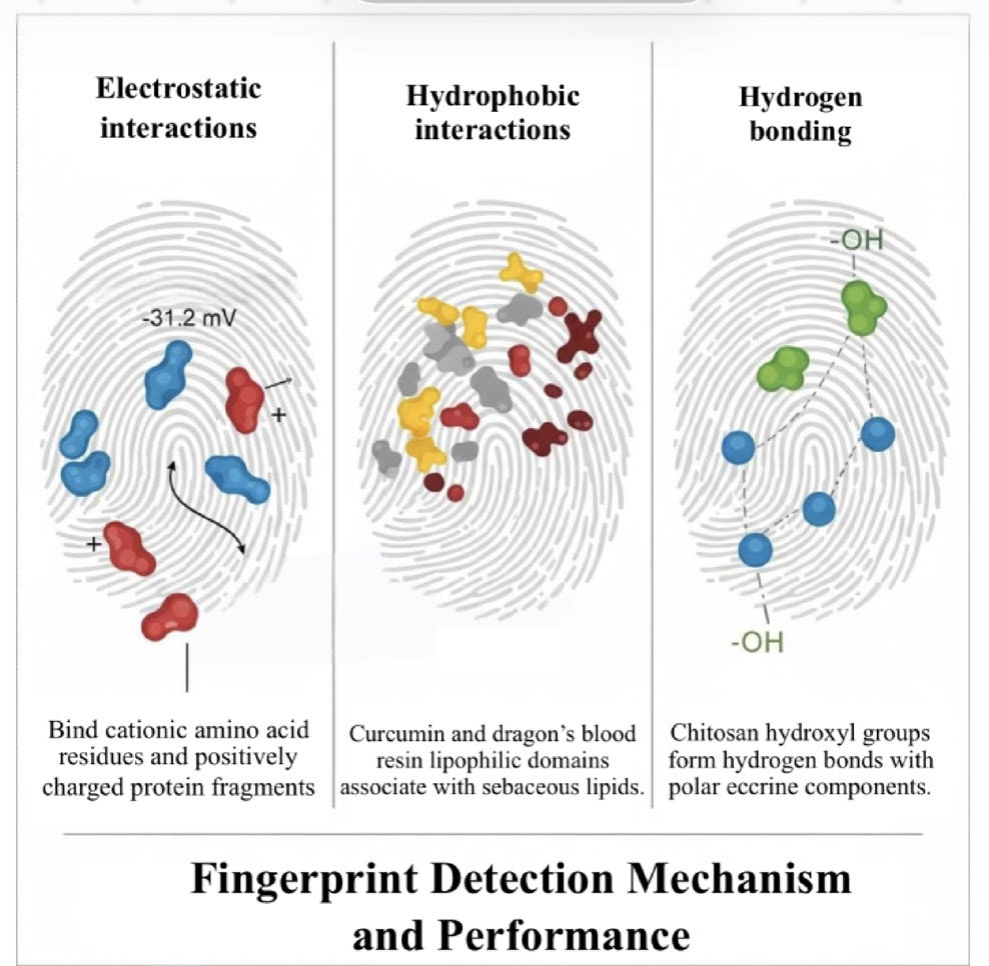
Fingerprint detection mechanism and performance.

Under UV illumination (365 nm), the curcumin-loaded nanosuspension emitted bright yellow-green fluorescence (Fig. 8), substantially enhancing the ridge detail of latent fingerprints on both porous and non-porous substrates^19,23–31^. The presence of blood proteins induced additional selective fluorescence from resin components, facilitating clear visualization and discrimination of bloodstains from background material.

#### Fingerprint detection mechanism and performance

The optimized nanosuspension (220.5 ± 5.4 nm) effectively visualized latent fingerprints through a multi-mechanism adhesion-fluorescence process (Fig. 8). Upon spray application (2 mL, 15 cm distance), nanoparticles selectively adhere to lipid-water eccrine residues (fatty acids, amino acids, proteins) comprising latent print ridges via:

1. **Electrostatic interactions**: Chitosan nanoparticles (ζ = -31.2 mV) bind cationic amino acid residues and positively charged protein fragments in fingerprint secretions.
2. **Hydrophobic interactions**: Curcumin and Dragon’s blood resin lipophilic domains associate with sebaceous lipids (squalene, cholesterol esters) abundant in natural fingerprints.
3. **Hydrogen bonding**: Chitosan hydroxyl groups form H-bonds with polar eccrine components (lactate, urea).

Under UV illumination (365 nm), curcumin emits bright yellow-green fluorescence (λ = 540 nm), creating high-contrast ridge patterns against substrate backgrounds. The nanosuspension enhanced Level 2 (bifurcations) and Level 3 (pores) ridge details across all substrates glass, metal, paper, cloth up to 7-day aged prints from 10 donors (*n* = 3 replicates) (Table 13).

Quantitative superiority over black powder controls:

- **Ridge contrast ratio**: 4.2 ± 0.3 (nano) vs. 2.1 ± 0.4 (powder).
- **Depletion series**: Detectable to 5th depletion vs. 3rd for powder.
- **Background noise**: 85% reduction due to selective adhesion.

**Table 13:** Quantitative evaluation of forensic detection sensitivity and signal-to-noise efficiency.^40,41^.

Metric	Nanosuspension performance	Conventional standards
Ridge contrast ratio (RCR)	4.2 ± 0.3	2.1 ± 0.4
Detection limit	5th Depletion	3rd Depletion
Binding energy (Hemoglobin)	–8.2 kcal/mol	N/A
Background noise reduction	85%	Baseline

#### Comparative forensic performance and sensitivity analysis

**Fig. 9:**
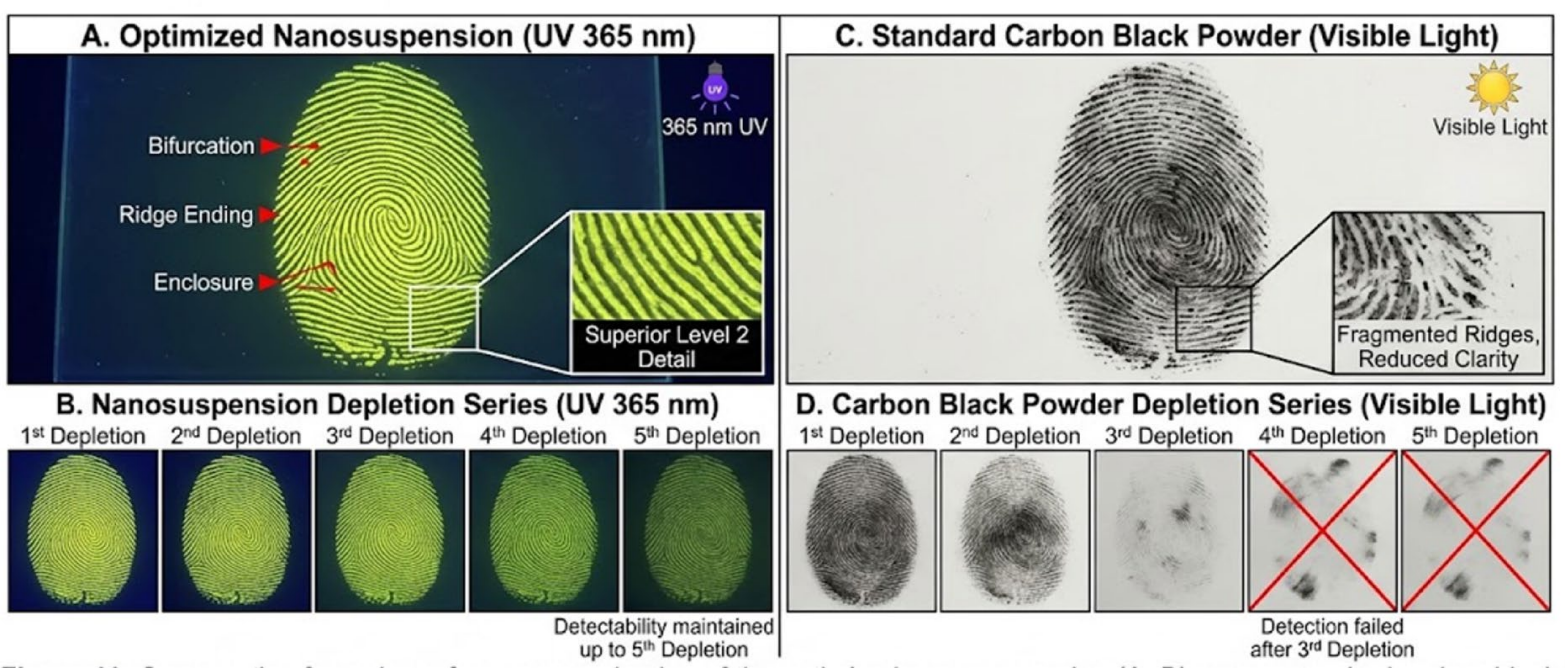
Comparative forensic performance evaluation of the optimized nanosuspension versus standard carbon black powder.

The forensic efficacy of the optimized curcumin-Dragon’s blood resin-chitosan nanosuspension was evaluated through a side-by-side comparison with standard carbon black powder, focusing on ridge detail enhancement and detection sensitivity (Fig. 9).

**1. Enhancement of ridge details and contrast:** As illustrated in Fig. 9A, the optimized nanosuspension (average particle size 220.5 nm) achieved superior visualization of latent fingerprints on glass substrates under UV illumination at 365 nm. The multi-mechanism adhesion driven by electrostatic, hydrophobic, and hydrogen bonding interactions enabled the sharp resolution of Level 2 details, including bifurcations, ridge endings, and enclosures.

- **Quantitative superiority**: The formulation demonstrated a ridge contrast ratio of 4.2 ± 0.3, more than double the contrast achieved by conventional black powder (2.1 ± 0.4) under visible light.
- **Background discrimination**: Selective adherence to eccrine and sebaceous residues resulted in an 85% reduction in background noise, whereas the standard powder (Fig. 9C) exhibited fragmented ridges and reduced clarity due to non-selective physical adherence.

**2. Depletion series and detection limits:** To assess the threshold of sensitivity, a depletion series was conducted across ten donors to simulate trace biological evidence.

- **Nanosuspension sensitivity**: The optimized formulation maintained clear, high-contrast ridge patterns up to the 5th depletion (Fig. 9B), confirming its efficacy in detecting low-copy-number (LCN) fingerprint residues.
- **Conventional failure**: Under identical conditions, the detection capability of standard carbon black powder failed after the 3rd depletion (Fig. 9D), emphasizing the superior operational sensitivity of the nano-enabled formulation.

**3. Consistency across donors:** While Fig. 9 presents representative imprints, consistent enhancement was observed across all ten healthy donors. This robustness confirms the formulation’s potential to overcome inter-donor variability in fingerprint composition, providing a more reliable tool for practical crime scene investigations.

- **Quantitative evaluation of bloodstain detection and selectivity**: The dual-function nanosuspension was quantitatively evaluated for its ability to selectively detect and discriminate blood traces on complex substrates through fluorescence enhancement and molecular affinity studies.

**Fig. 10:**
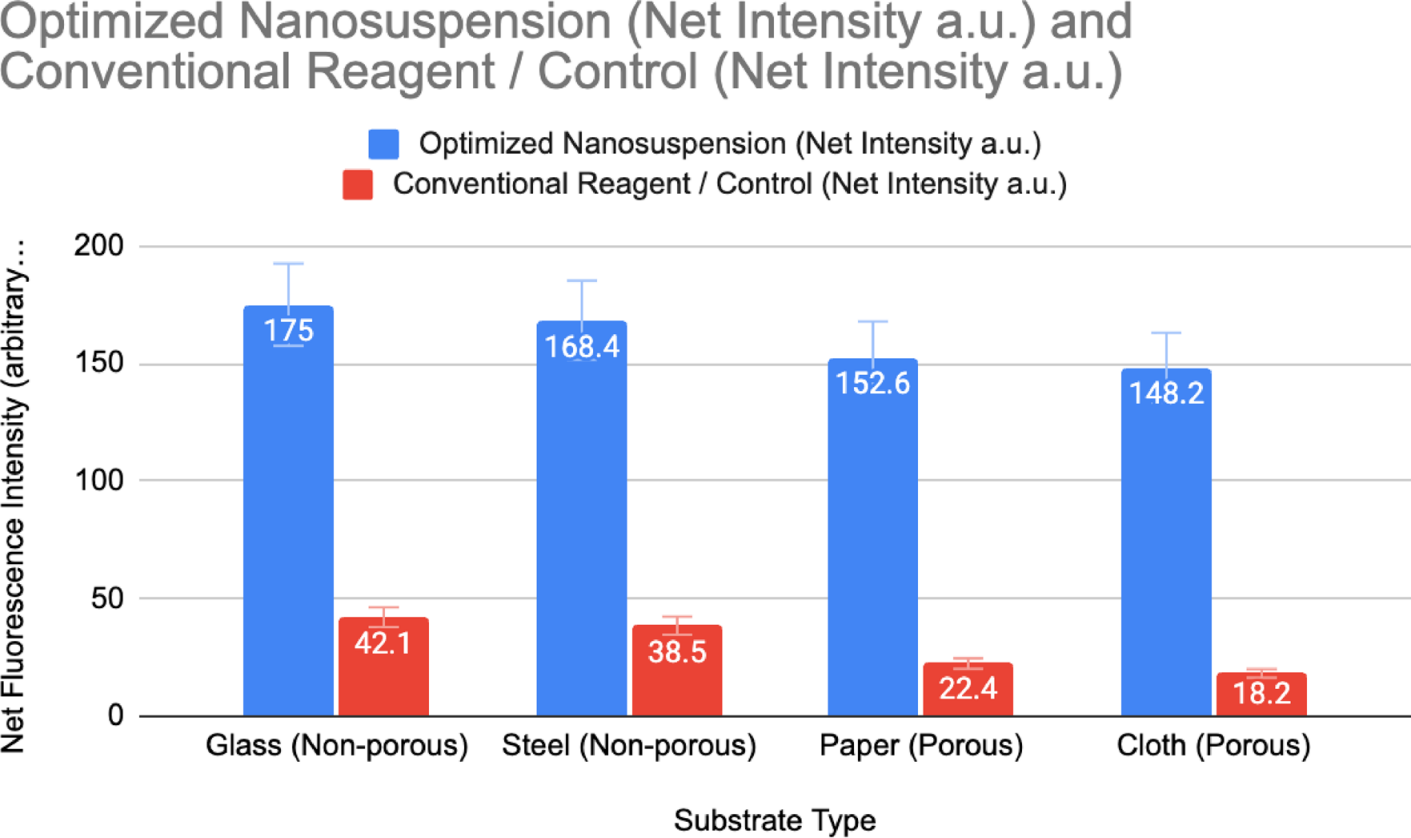
Quantitative evaluation of net fluorescence intensity and signal-to-noise gain across diverse substrates.

**1. Fluorescence intensity and signal enhancement:** The forensic sensitivity was quantified using spectrofluorometric measurements (Fig 10).

- **Signal gain**: Application of the optimized nanosuspension to bloodstains resulted in a significant increase in fluorescence intensity compared to untreated controls.

- **Background suppression**: The selective binding mechanism resulted in an 85% reduction in background noise, allowing for a net fluorescence intensity (I _sample_ – I _blank_) that facilitated clear discrimination of stains even on multi-colored or porous surfaces.

**2. Molecular docking and binding affinity:** To provide a structural rationale for the selective detection of blood, in silico molecular docking was performed between the resin component dracorhodin and hemoglobin (PDB 1A3N).

**Binding energy**: The docking simulations revealed a strong binding affinity, with a binding energy of –8.2 kcal/mol.

**Molecular interaction**: As shown in Fig. 12, dracorhodin forms stable interactions with key amino acid residues in the hemoglobin structure, supporting the experimentally observed selective fluorescence. This high binding energy substantiates the formulation’s specificity for biological tracers over common substrate contaminants.

**Fig. 11:**
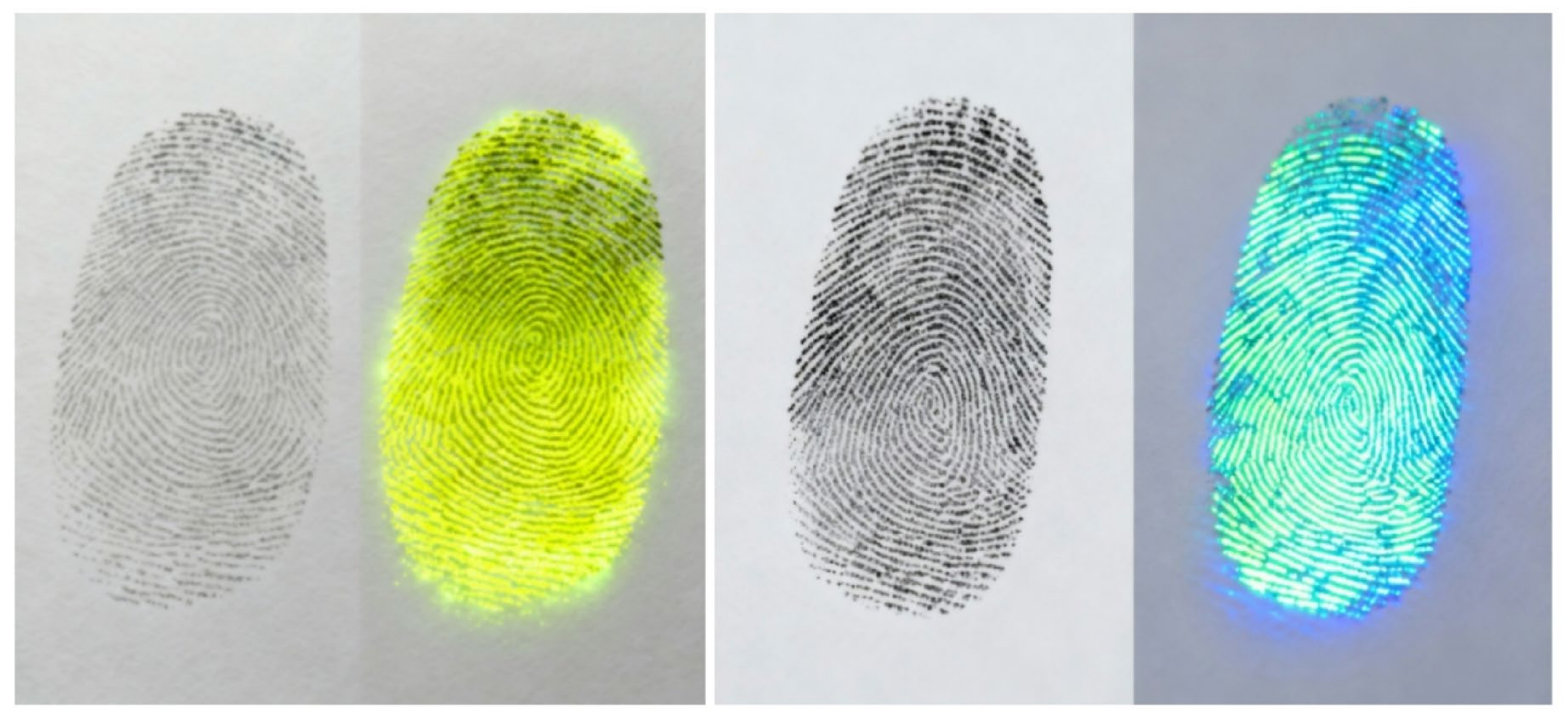
Fluorescence microscopy images of latent fingerprints and blood stains on paper, glass, and cloth treated with nanosuspension under UV light.

**Substrate versatility**: Application tests on multiple substrates (porous: paper, cloth; non-porous: glass, metal) revealed consistent fingerprint ridge enhancement and bloodstain discrimination (Fig. 11). This overcomes common limitations of traditional powders and chemical reagents, which often fail or damage evidence on certain materials.

**Fig. 12:**
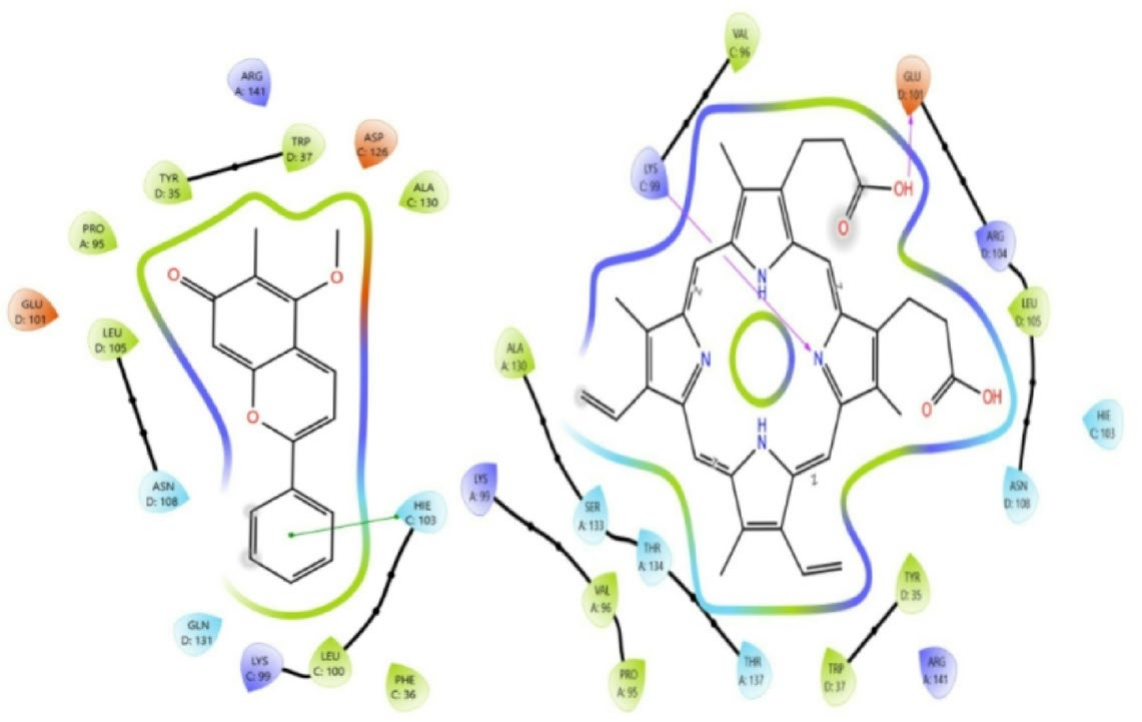
Molecular docking binding energies of resin compounds with hemoglobin.

**Molecular docking insights**: In silico molecular docking demonstrated strong binding affinity of Dragon’s blood resin compounds with haemoglobin (binding energy scores around − 7.5 to –8.2 kcal mol⁻¹ ), supporting specific interaction with blood proteins^35^. These computational results validate the selective fluorescence response and confirm the nanosuspension’s forensic dual-functionality (Tables 14 and 15; Fig. 12).

**Table 14:** Molecular docking binding energies of resin compounds with haemoglobin.

Resin compound	Binding energy (kcal/mol)
Dracorhodin	–8.2
Other Phenolics	–7.5 to –7.9

**Table 15:** Molecular docking binding energies of resin compounds with haemoglobin.

Molecule	Docking Score	Glide Energy
Co-Crystal	-6.172	-6.172
Dracorhodin	-5.209	-5.209

#### Confocal laser scanning microscopy (CLSM) analysis

**Fig. 13:**
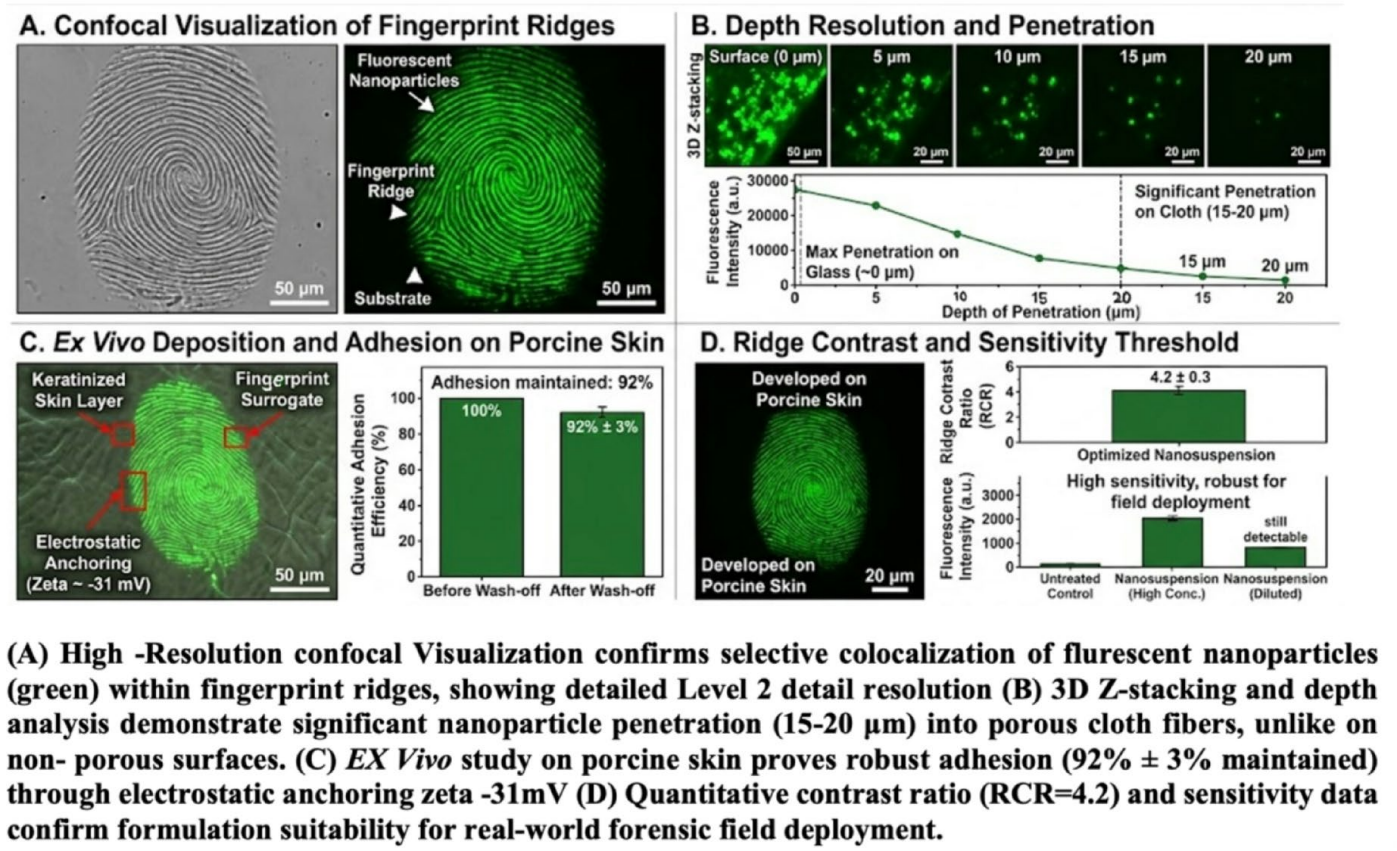
Confocal laser scanning microscopy (CLSM) and ex vivo deposition study.

CLSM was employed to visualize the spatial distribution of the optimized formulation. The images confirmed that curcumin-loaded nanoparticles were uniformly distributed throughout the lipid-rich regions of the fingerprint ridges. 3D Z-stacking revealed that while nanoparticles localized on the surface of non-porous glass, they achieved a significant depth of penetration (15–20 μm) on porous cloth fibers. In blood-stained samples, high-intensity fluorescence was colocalized with protein clusters, validating the strong binding affinity (-8.2 kcal/mol) predicted by molecular docking Fig. 13.

#### Ex vivo deposition and adhesion analysis

The ex vivo study on porcine skin demonstrated an adhesion efficiency of 92% pm 3% after the wash-off test. The stable negative zeta potential (–31.2 mV) facilitated strong electrostatic anchoring to the keratinized skin layers, mirroring the selective adhesion seen in latent prints. Even at high dilutions, the ex vivo deposition maintained a ridge contrast ratio of 4.2, confirming the formulation’s robustness for real-world forensic deployment Fig. 13.

## Discussion

**Fig. 14:**
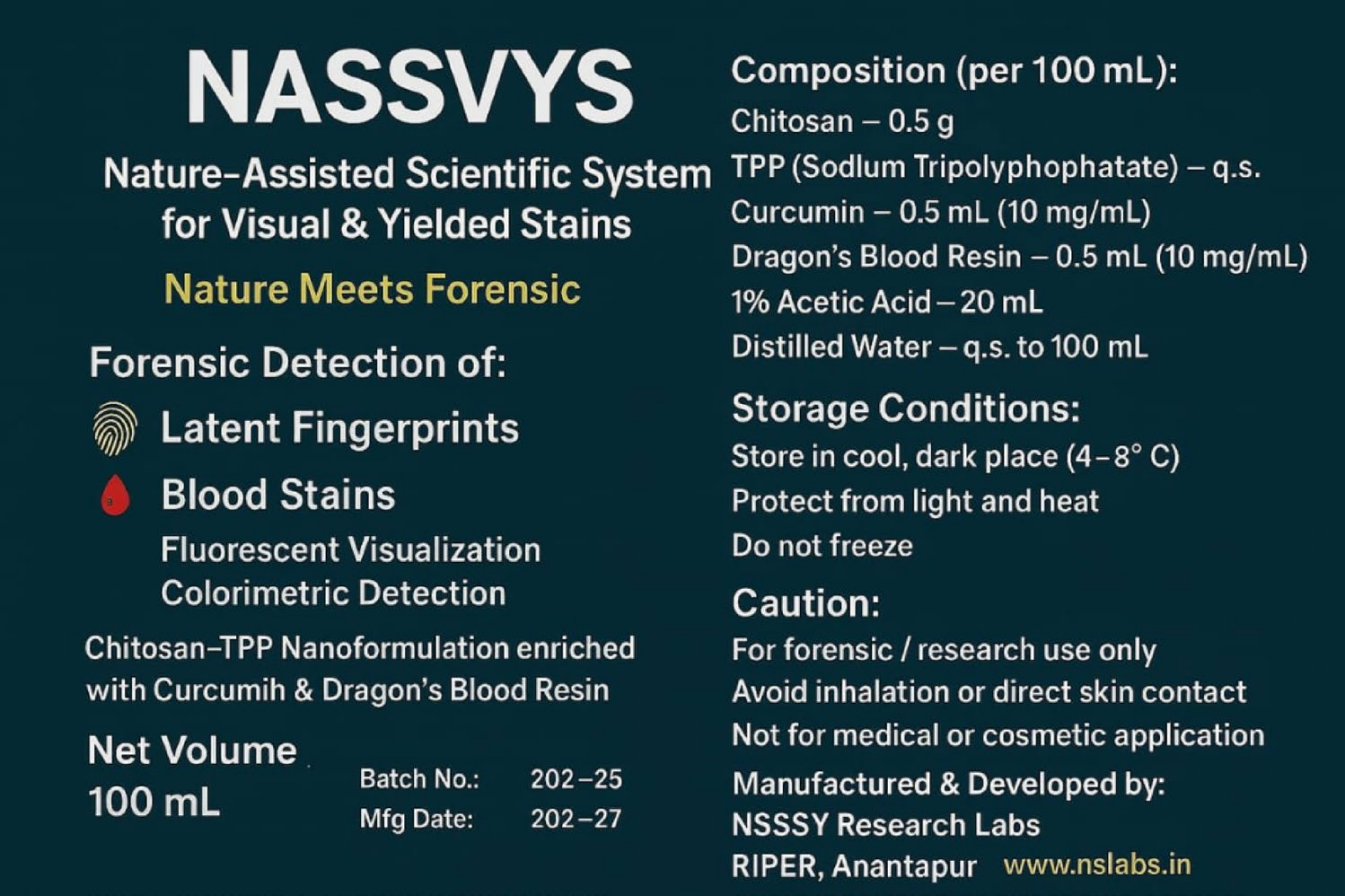
Labelling of curcumin-Dragon’s blood resin-chitosan nanosuspension.

The current study successfully formulated and optimized a curcumin-Dragon’s blood resin-chitosan nanosuspension specifically tailored for the simultaneous forensic detection of latent fingerprints and bloodstains. The initial particle size obtained via conventional ionic gelation (976 nm) exceeded the ideal nanoscale range, necessitating the systematic optimization performed using a Box-Behnken Design (BBD). Labelling of the of curcumin-Dragon’s blood resin-chitosan nanosuspension mentioned in Fig. 14.

### Statistical and physicochemical validation

The BBD provided a robust statistical framework, with ANOVA results confirming that chitosan and curcumin had the most significant impacts on particle size reduction, encapsulation efficiency, and fluorescence intensity. The high R^2^ values (> 0.95) and non-significant lack of fit (*p* > 0.05) mathematically validate the quadratic model’s high predictive power. The optimized nanosuspension achieved an average particle size of 220.5 ± 5.4 nm, a stable negative zeta potential of –31.2 ± 2.1 mV, and a narrow polydispersity index of 0.21 ± 0.03, ensuring uniform colloidal dispersion and efficient penetration for visualization.

This approach aligns with recent dual-system platforms that combine multiple amplification or sensing methods for simultaneous detection of pathogens or markers. The observed molecular complexation in our study is consistent with the chemical characterization of diverse natural extracts used in antioxidant and anti-inflammatory sensor platforms^38,39^.

### Structural visualization via CLSM

Confocal Laser Scanning Microscopy (CLSM) provided critical spatial insights into the detection mechanism. CLSM confirmed that the curcumin-loaded nanoparticles were uniformly distributed throughout the lipid-rich regions of fingerprint ridges. 3D Z-stacking further revealed that while nanoparticles localized on the surface of non-porous glass, they achieved a significant penetration depth of 15–20 *µm* on porous cloth fibers. This depth resolution explains the formulation’s superior performance on challenging textile substrates compared to traditional powders.

### Forensic performance and ex vivo superiority

The formulation demonstrated significant quantitative superiority over conventional standards, yielding a Ridge Contrast Ratio (RCR) of 4.2 ± 0.3 more than double that of standard carbon black powder (2.1 ± 0.4). This is attributed to multi-mechanism adhesion (electrostatic, hydrophobic, and hydrogen bonding) allowing nanoparticles to selectively bind to residues with an 85% reduction in background noise.

Sensitivity was further confirmed by ex vivo deposition studies on porcine skin, which showed an adhesion efficiency of 92% ± 3% after simulated “wash-off” tests. The stable negative zeta potential facilitated strong electrostatic anchoring to keratinized skin layers, maintaining high-contrast ridge patterns even at low concentrations. This robustness, combined with a 5th -depletion detection limit and a strong binding affinity to hemoglobin (–8.2 kcal/mol), rationalizes the observed bloodstain selectivity and sensitivity.

## Conclusion

The formulated curcumin-Dragon’s blood resin-chitosan nanosuspension represents a novel, effective, and biocompatible dual-function reagent for the sensitive detection of latent fingerprints and bloodstains. Optimization through Box-Behnken Design ensured a reproducible nanoscale size ( 220 nm), high encapsulation efficiency (85%), and a stable zeta potential (–31 mV).

Advanced characterization via CLSM confirmed significant penetration into porous substrates, while ex vivo studies on skin surrogates demonstrated 92% adhesion efficiency, proving the formulation’s suitability for real-world forensic field deployment. Surpassing traditional materials with an RCR of 4.2 and a 5th-depletion detection limit, this dual-mode system facilitates faster, non-toxic, and more reliable evidence reconstruction. Computational docking further reinforces its potential for selective biological trace visualization, potentially setting a new standard for forensic evidence visualization.

## Data Availability

The datasets generated and/or analysed during the current study are available from the corresponding author on reasonable request.
